# Neurochemical Plasticity of the Coeliac-Superior Mesenteric Ganglion Complex Neurons Projecting to the Prepyloric Area of the Porcine Stomach following Hyperacidity

**DOI:** 10.1155/2016/8596214

**Published:** 2016-05-15

**Authors:** Katarzyna Palus, Jarosław Całka

**Affiliations:** Department of Clinical Physiology, Faculty of Veterinary Medicine, University of Warmia and Mazury in Olsztyn, 10-719 Olsztyn, Poland

## Abstract

This study was designed to determine neurochemical properties of the coeliac-superior mesenteric ganglion (CSMG) neurons supplying the prepyloric area of the porcine stomach in physiological state and following experimentally induced hyperacidity. To localize sympathetic neurons innervating the studied area of stomach, the neuronal retrograde tracer Fast Blue (FB) was applied to control animals and hydrochloric acid infusion (HCl) groups. After 23 days, animals of the HCl group were reintroduced into a state of general anesthesia and intragastrically given 5 mL/kg of body weight of 0.25 M aqueous solution of hydrochloric acid. On the 28th day, all animals were sacrificed. The CSMG complexes were then collected and processed for double-labeling immunofluorescence. In the control animals, FB-positive perikarya displayed immunoreactivity to tyrosine hydroxylase (TH), dopamine *β*-hydroxylase (D*β*H), neuropeptide Y (NPY), and galanin (GAL). Experimentally induced gastric hyperacidity changed the neurochemical phenotype of the studied neurons. An upregulated expression of GAL and NPY and the* de novo* synthesis of neuronal nitric oxide synthase (nNOS) and leu5-enkephalin (LENK) as well as downregulated expression of TH and D*β*H in the stomach-projecting neurons were observed. These findings enrich existing knowledge about the participation of these active substances in adaptive mechanism(s) of the sympathetic neurons during pathological processes within the gastrointestinal tract.

## 1. Introduction

Gastrointestinal disorders, especially acid-related diseases, including peptic and duodenal ulcers, gastroesophageal reflux disease, upper GI bleeding, or stress-related mucosal disease, are currently serious health issues encountered very frequently in patients worldwide [1]. On the other hand, many gastrointestinal diseases, such as Zollinger-Ellison syndrome, retained antrum syndrome, antral G cell hyperplasia, and gastric outlet obstruction, are disorders whose etiology involves gastrin hypersecretion [2–4]. It has been shown that gastrin, histamine, and acetylcholine stimulate gastric acid secretion, while somatostatin, cholecystokinin, glucagon-like peptide-1, and atrial natriuretic peptide reduce secretory stomach activity [5].

The mechanisms of the above-mentioned pathological states are not quite clear, but disturbances in the gastric secretion of hydrochloric acid seem to be one of the main causes. For example, the fundamental role of hyperacidity has been proven in the development of gastric ulcerative disease [3]. One theory suggests that ulcers are the result of an imbalance between aggressive factors (acid, pepsin,* Helicobacter pylori*, and nonsteroidal anti-inflammatory drugs) and local mucosal defensive factors (mucous bicarbonate, blood flow, and prostaglandins) leading to active local inflammation [6]. On the other hand, many aspects connected with gastric acid-related diseases remain obscure. One of them is the function of the nervous system supplying the gastrointestinal (GI) tract during pathological processes.

In view of previous studies, the gastric mucosa is innervated by both enteric nervous system (ENS) and extrinsic sympathetic and sensory innervation [7–9]. Gastric sympathetic nerve fibers, which enter the stomach wall along arteries, can derive from different nervous structures, depending on the animal species. Previous studies on rodents based on retrograde tracing have shown that the sympathetic fibers supplying the stomach mucosa are derived mostly from paravertebral ganglia and, to a lesser extent, from the celiac ganglion (CG) [10–12]. In turn, the main source of the sympathetic output to the abdominal viscera in pigs arises from prevertebral ganglia and, to a minor extent, derives from the sympathetic chain ganglia (SchG) [13, 14]. The sympathetic nervous system (SNS) supplying the GI tract participates in the control of motility, secretion, and vasoregulation and also modulates gastric and intestinal inflammatory processes [13, 15]. These functions are performed with the help of a very broad spectrum of neuronal active substances, which include, besides the main sympathetic neuromediators—noradrenaline and other neuromediators and/or neuromodulators—such as neuropeptide Y (NPY), Met5-enkephalin-Arg6-Gly7-Leu8 (MEAGL), somatostatin (SOM), vasoactive intestinal polypeptide (VIP), and galanin (GAL) [16].

It is also important to note that various pathological processes within the GI tract, such as inflammation or neuronal damage, may trigger a plastic response in enteric nervous system as well as extrinsic sympathetic intestinal innervation and the main symptom of this response is a change in the expression of neuronal active substances [8, 14, 17]. However, to date, the response of sympathetic neurons to gastric mucosal injury and local inflammation following hyperacidity is unknown. Thus, the present study was designed to determine the neurochemical properties of the CSMG neurons supplying the prepyloric area of the porcine stomach in a physiological state and following experimentally induced hyperacidity by using combined retrograde tracing and double-labeling immunohistochemistry. The choice of the domestic pig as an experimental model in the present study is not accidental and is justified by the high degree of physiological and anatomical similarity to human digestive system functions, in contrast to small animals, especially rodents [18, 19].

## 2. Materials and Methods

### 2.1. Animals and Experimental Procedures

Ten juvenile female pigs of the Large White Polish breed (aged approx. 8 weeks and of 20 kg body weight) were used in this experiment. All animals were kept under standard laboratory conditions, fed a commercial grain mixture, and had free access to water. All experimental procedures were in accordance with the rules of the Local Ethical Committee for Experiments on Animals in Olsztyn (decision number 05/2010).

On the first day of experiment, median laparotomy to expose the prepyloric areas of stomach was performed in all pigs under general anesthesia induced by azaperone (Stresnil, Jansen Pharmaceutica N.V., Belgium, 4 mg/kg of body weight, i.m.) and sodium thiopental (Thiopental, Sandoz, Kundl-Rakusko, Austria; 10 mg/kg of body weight, i.v.). A total volume of 50 *µ*L (1 *µ*L per 1 injection) of a 5% aqueous solution of the fluorescent retrograde neuronal tracer Fast Blue (FB, EMS-CHEMIE, GmbH, Germany) was then applied to the diamond-shaped part (ca. 4 cm × 4 cm) of the stomach anterior prepyloric wall (located about 1 cm from the greater curvature of the stomach and 3 cm from the pylorus).

Afterwards, the pigs were divided into two experimental groups: control group (C group, *n* = 5) and animals with hydrochloric acid infusion (HCl group, *n* = 5). After 23 days, animals of the HCl group were reintroduced into a state of general anesthesia (as described above) and intragastrically given 5 mL/kg of body weight of a 0.25 M aqueous solution of hydrochloric acid using a stomach tube. Gastroscopic examination under general anesthesia (using a video-endoscope Olympus GIF 145 with a 1030 mm working length and 9.8 mm diameter) was performed to exclude lesions in the gastric mucosa in animals from the HCl group in the first day and to confirm pathological changes caused by experimentally induced hyperacidity one week after hydrochloric acid infusion. Directly after gastroscopy, animals of the HCl group were sacrificed by an overdose of sodium thiopental. On the same day, animals of the control group were also reanaesthetized and euthanized by an overdose of sodium thiopental. All animals were then transcardially perfused with 4% buffered paraformaldehyde (pH 7.4) and the coeliac-superior mesenteric ganglion complexes (CSMG) were collected and postfixed by immersion in the same fixative for 20 minutes, rinsed in phosphate buffer (pH 7.4) for three days, and then stored in a 30% buffered sucrose solution until sectioning.

### 2.2. Immunohistological Procedures

The CSMG complexes were cut with a cryostat (Microm HM-525, at −22°C) into 14 *μ*m thick serial sections and analyzed with an Olympus BX 51 fluorescent microscope (Olympus, Poland) equipped with a filter set suitable for observation of FB to localize and count neurons containing the tracer. To establish the number of FB-positive perikarya, neurons were counted in every fourth section. Only neurons with a clearly visible nucleus were considered. The sections were then subjected to routine double-labeling immunofluorescence. The sections were briefly air-dried at room temperature for 45 min and rinsed in 0.1 M phosphate-buffered saline (PBS, pH 7.4; 3 × 10 min). Subsequently, these sections were blocked with a mixture containing 10% horse serum and 0.1% bovine serum albumin in 0.1 M PBS, 1% Triton X-100, 0.05% Thimerosal, and 0.01% sodium azide for 1 h at room temperature, rinsed in PBS (3 × 10 min), and incubated overnight with primary antisera raised against the tyrosine hydroxylase (TH) (mouse, cat. number MAB 318, Millipore, USA, working dilution 1 : 200) and dopamine *β*-hydroxylase (D*β*H) (rabbit, cat. number AB1585, Millipore, USA, working dilution 1 : 500), NPY (rabbit, cat. number NA1115, Biomol, Germany, working dilution 1 : 1000), GAL (rabbit, cat. number AB2233, Millipore, USA, working dilution 1 : 2000), neuronal nitric oxide synthase (nNOS) (rabbit, cat. number AB5380, Millipore, USA, working dilution 1 : 2500), and leu5-enkephalin (LENK) (rabbit, cat. number 4140-0355, AbD Serotec, UK, working dilution 1 : 500). Following subsequent rinsing in PBS (3 × 10 min), the sections were incubated with species-specific secondary antibodies conjugated to Alexa Fluor 488 (donkey anti-mouse IgG, cat. number A21202, Invitrogen, USA, working dilution 1 : 1000) and Alexa Fluor 546 (goat anti-rabbit IgG, cat. number A11010, Invitrogen, USA, working dilution 1 : 1000) for 1 h at room temperature. Next, the washed sections were cover-slipped in carbonate-buffered glycerol (pH 8.6). The negative control included a preabsorption test for the primary antisera (20 *μ*g specific antigen/mL of the tested serum; all antigens were manufactured by Peninsula, Sigma, or AbD Serotec), an omission test, and a replacement test (replacement of primary antisera with the corresponding nonimmune sera). These procedures completely eliminate specific stainings.

The labeled perikarya were evaluated under the Olympus BX51 microscope equipped with epifluorescence and appropriate filter sets and photographed with a digital camera connected to a PC and processed with Olympus Cell F image-analysis software (Olympus, Tokyo, Japan). To determine the percentage of the particular neuronal subpopulations, at least 200 FB-positive neurons per animal were studied, in sections separated by at least 100 *µ*m.

### 2.3. Statistical Procedures

The mean (±standard error of mean (SEM)) number of FB-labeled neurons immunoreactive to the particular investigated antibody was analyzed using Statistica 10 software (StatSoft Inc., Tulsa, OK, USA). The differences between the control and HCl group were evaluated using Student's *t*-test for independent samples and considered to be significant at ^*∗*^ *P* < 0.05 and very significant at ^*∗∗*^ *P* < 0.001.

## 3. Results

### 3.1. Gastroscopic Examination

**Figure 1:**
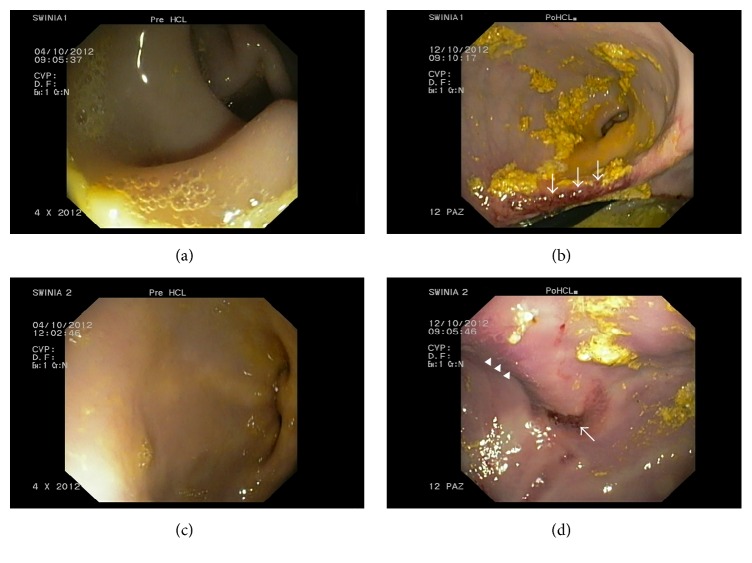
The gastric mucosa without lesions before HCl infusion (a), (c). Macroscopic changes in the gastric mucosa caused by hyperacidity: (b) deep erosion (arrows), (d) hyperaemia (arrows heads), and superficial erosion (arrow).

The gastroscopic examination performed on the first day of the experiment excluded lesions in the gastric mucosa in animals of the HCl group. The same examination performed one week after hydrochloric acid infusion revealed macroscopic changes in the gastric mucosa, such as petechia, erosions, and hyperaemia, which confirmed inflammation caused by hyperacidity (Figures 1(a), 1(b), 1(c), and 1(d)).

### 3.2. Distribution of FB+ Neurons in the Control and Experimental Group

**Figure 2:**
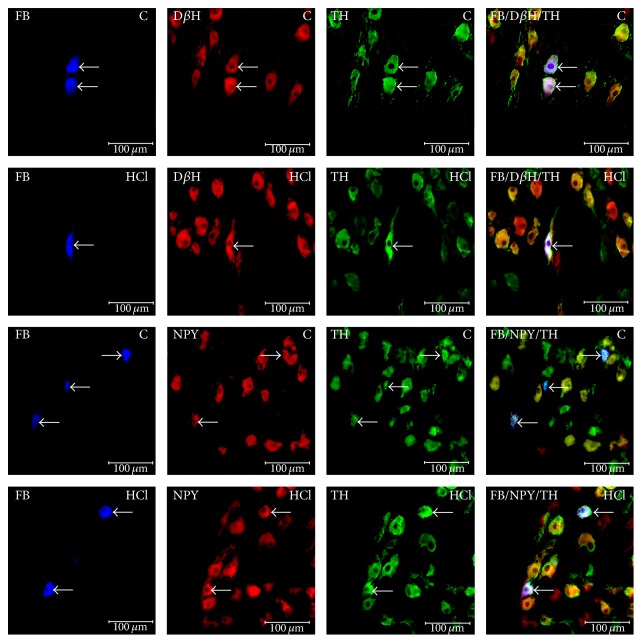
Fast Blue-positive neurons (FB) displaying immunoreactivity to tyrosine hydroxylase (TH) and dopamine *β*-hydroxylase (D*β*H) or neuropeptide Y (NPY) in control animals (C) contrary to animals of HCl group (HCl). Right column has been created by digital superimposition of three colour channels.

**Figure 3:**
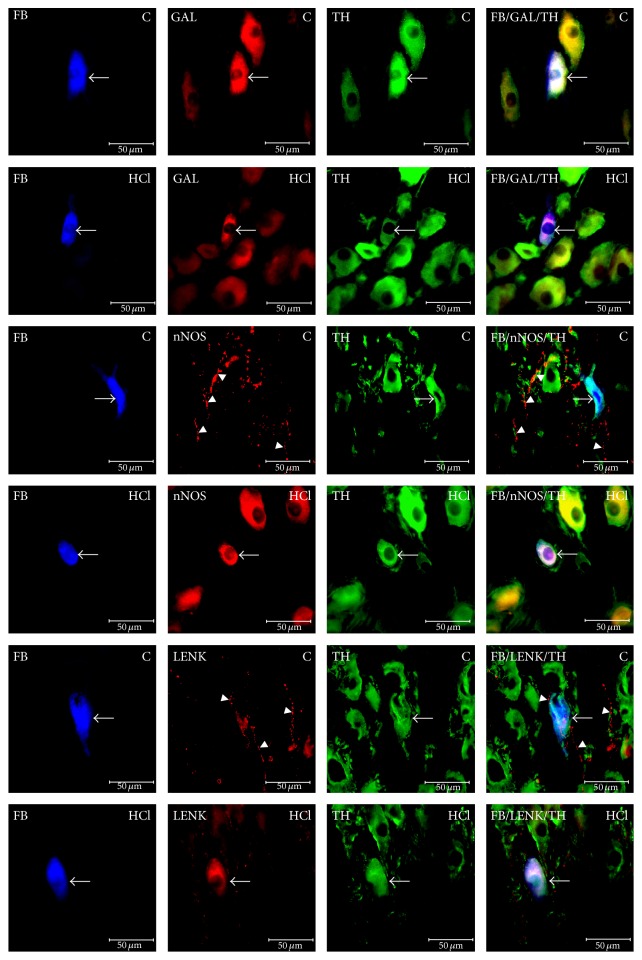
Fast Blue-positive neurons (FB, arrows) immunoreactive to galanin (GAL) and nerve fibers (arrows heads) displaying immunoreactivity to neuronal nitric oxide synthase (nNOS) and leu5-enkephalin (LENK) in control animals (C) contrary to animals of HCl group (HCl). Right column has been created by digital superimposition of three colour channels.

The CSMG neurons innervating the prepyloric area of the porcine stomach were localized exclusively within the region of coeliac poles of the CSMG complex. All FB-positive neurons in both the control and experimental groups were similar in morphology. FB+ cells were round, oval, and multipolar in shape, with a centrally situated nucleus, and 20–45 *µ*m in diameter. The labeled neurons (FB-positive cells) were scattered throughout the CSMG complex but rarely formed clusters of 2–5 cells in the microscopic observation field. After experimentally induced hyperacidity, the total number of gastric neurons in the CSMG changes only in a statistically insignificant manner compared to the control ganglia (1655 ± 36.6 versus 1615 ± 20.73, resp.) (Figures 2 and 3).

### 3.3. Immunohistochemical Characteristics of FB+ Neurons in Control Animals

Immunohistochemistry revealed that the vast majority of the retrograde-labeled neurons displayed immunoreactivity (IR) to TH (94.85 ± 1.01%) (Figures 2 and 3). Among the FB+/TH+ nerve cells, many were immunoreactive to D*β*H (97.10 ± 0.97%) (Figure 2). The other substances found in FB+/TH+ neurons were NPY (46.88 ± 2.53%) (Figure 2) and/or GAL (8.40 ± 0.53%) (Figure 3). However, FB+/TH+ neurons were immunonegative to nNOS and LENK and FB+/TH+ perikarya were supplied with varicose nerve fibers exhibiting immunoreactivities to nNOS (Figure 3) and LENK (Figure 3).

### 3.4. Immunohistochemical Characteristics of FB+ Neurons after Experimentally Induced Hyperacidity

Experimentally induced gastric hyperacidity did not change the number of FB-positive neurons. In contrast, clear differences in the expression of particular active substances were observed. Microscopic examination showed that the CSMG neurons supplying the prepyloric area of the porcine stomach responded to gastric hyperacidity by changes in their chemical coding. In the FB+ neurons, hyperacidity was followed by a visible decrease in TH-immunoreactivity (86.00 ± 2.42% versus 94.85 ± 1.01%; ^*∗*^ *P* < 0.05) (Figure 2). A lower number of FB+/TH+/D*β*H+ neurons (89.88 ± 1.84% versus 97.10 ± 0.97%; ^*∗*^ *P* < 0.05) (Figure 2) were also observed. On the other hand, an increase in the number of FB+/TH+/NPY+ (57.83 ± 2.04 versus 46.88 ± 2.53%; ^*∗*^ *P* < 0.05) (Figure 2) and FB+/TH+/GAL+ (22.52 ± 1.18% versus 8.40 ± 0.53%; ^*∗∗*^ *P* < 0.001) (Figure 3) neurons was encountered. Additionally, FB+/TH+ perikarya displayed immunoreactivity to nNOS (8.18 ± 0.75%; ^*∗∗*^ *P* < 0.001) (Figure 3) and LENK (5.57 ± 0.43%; ^*∗∗*^ *P* < 0.001) (Figure 3), even though the labeled neurons in the control group did not contain these active substances. Furthermore, the density of nNOS- and LENK-IR nerve fibers running near the studied neurons was reduced compared to the control group.

## 4. Discussion

The present experiment has demonstrated that the CSMG complex is a significant source of the sympathetic innervation of the porcine stomach. To date, such studies have been mainly performed on rats, guinea pigs, cats, and dogs [7, 10, 20–23]. Thus, the present study, for the first time, describes the morphology, distribution, and chemical coding of the CSMG neurons supplying the porcine stomach.

The obtained results show that nearly all the stomach-projecting CSMG neurons are noradrenergic (being simultaneously immunoreactive to TH and D*β*H). This observation corresponds to the results of previous studies [10, 12] dealing with the contribution of the noradrenergic neurons in the gastrointestinal tract innervation. This is supported by the findings of Łakomy et al. [16] indicating the noradrenergic character of the porcine CSMG neurons. Interestingly, Paton and Vizi [24] have demonstrated that noradrenergic innervation controls gastrointestinal movement through presynaptic inhibitory effects on cholinergic neurons. Additionally, many of noradrenergic FB-positive neurons exhibited immunoreactivity to NPY and GAL. These active substances were also found in sympathetic neurons supplying the gastrointestinal tract, although immunoreactivity to NPY and GAL clearly depend on both the animal species and the fragment of the GI tract studied [25, 26]. The above data, together with the results of this experiment, may suggest that TH, D*β*H, NPY, and GAL are involved in the sympathetic regulation of digestive functions.

Experimentally induced hyperacidity caused a pronounced effect on the immunoreactivity of CSMG neurons supplying the stomach. In general, the increase in the population of neurons immunoreactive to NPY and GAL, the decrease in the number of neurons immunopositive to TH and D*β*H, and the* de novo* synthesis of nNOS and LENK have been demonstrated. The nature of the observed changes depended on the substance tested, which confirmed earlier reports that various substances play a variety of roles in sympathetic neurons [13, 16, 25, 27].

During the present study, the most visible changes between control and experimental animals pertained to the expression of GAL. Previous studies have shown that the function of galanin in the gastrointestinal tract is associated with the animal species and the part of the GI tract studied [28–30]. The increased expression of GAL observed in this study confirms previous reports findings about the participation of GAL in the regulation of pathological processes within the digestive tract. Notably, the enhanced expression of GAL has been observed in animal models of inflammation, including proliferative enteropathy [14], chemically induced colitis [13, 30],* Salmonella* infection [31], and gastritis caused by long-term aspirin administration [32, 33]. Furthermore, it has been demonstrated that galanin has an anti-inflammatory effect and is involved in the regulation of the secretion of proinflammatory cytokines such as TNF-*α*, IL-1*α*, and IL-8 [34]. Furthermore, in the pathogenesis of inflammatory bowel disease, both GAL and hormones are involved in the communication between the autonomous nervous system and the neurons of the enteric nervous system (ENS) [35, 36].

Interestingly, the present study has revealed that hyperacidity led to the* de novo* synthesis of nNOS in noradrenergic stomach-projecting neurons. It appears that this increased expression is due to the participation of nitric oxide in the defense mechanisms of gastric mucosa by affecting mucus secretion, repair of ulceration, maintenance of mucosal blood flow, and the activity of various mucosal immunological cells [37, 38]. Moreover, it should be emphasized that nitric oxide synthesized by nNOS has the ability to reduce inflammatory reactions of the gastrointestinal tract and protect the mucosa from injury [39]. These findings are in agreement with the results of other authors [32, 40], who also found a significant increase in the number of nNOS-IR neurons during inflammatory processes within the GI tract. In turn, the* de novo* synthesis of LENK in retrogradely labeled perikarya may suggest that this opioid peptide plays a similar function in the porcine stomach to that previously described in rodents, such as participation in nociception modulation, inhibition of the local inflammatory pain, and gastrin secretion [41]. It should also be emphasized that LENK modulates the release of other peptides in the gastrointestinal tract [42]. The results of this experiment suggest that LENK plays a role in porcine stomach hyperacidity pathology, although its exact function needs further study.

Considering the enhancement in NPY expression, it should be mentioned that the character and the exact mechanism of these changes are not fully elucidated. In general, NPY is considered an important neuronal and hormonal factor regulating gastrointestinal tract activity, such as gastrointestinal motility, gastric acid secretion, and exocrine function of the pancreas [43, 44]. A literature analysis showed that the increased expression of NPY was observed during various pathological conditions within the gastrointestinal tract, such as chronic inflammatory bowel disease [45], colitis [13], and proliferative enteropathy [14], which may suggest its modulating effect on inflammation and potentially neuroprotective functions. NPY is also involved in the control of inflammation by the recruitment of immature dendritic cells and in promoting helper T-cell polarization [46, 47]. This appears to be consistent with studies confirming the role of NPY in mediating between the nervous and immune system in the gastrointestinal tract [47]. On the other hand, in response to axotomy, both a downregulation [48] and an upregulation [49] of the NPY expression in sympathetic neurons have been observed, which may suggest that it depends on the species used or the innervated area of GI tract. However, the mechanism of these changes remains unexplained.

The present study also found a decrease in the expression of TH and D*β*H in CSMG complex neurons supplying the porcine stomach. This observation seems to be consistent with report of Vizi [50] who demonstrated inhibitory action of noradrenaline on acetylcholine release. Thus reduction of the catecholaminergic stomach input found in the present study may result in acetylcholine and consequently gastrin release. This observation supports the findings of Yamada et al. [51], who demonstrated similar changes in rodent sympathetic neurons. It can therefore be assumed that sympathetic neurons decrease the expression of active substances abundantly synthesized in a physiological state in order to enhance the expression of those involved in neuronal protection [13]. Furthermore, reduced expression of the physiological neurotransmitter was observed as a result of the secretion of inflammatory mediators, such as IL 1*β* and TGF-*β* [49]. Interestingly, NPY has an inhibitory effect on noradrenergic transmission, which is in line with the enhanced expression of this peptide observed in this study.

Although further efforts are needed to fully recognize the mechanism(s) of the observed changes in the course of hyperacidity, the changes in expression observed in this experiment may be due to changes at the axonal transport stage (inhibition of transport). This hypothesis can be supported by* de novo* synthesis of nNOS and LENK as well as a reduction of nNOS-IR and/or LENK-IR fiber density in the experimental group animals. Another possible mechanism which should be taken into account while discussing the present results is increased synthesis at various stages of this process (transcription, translation, or changes in the activity of enzymes involved in the synthesis), which may confirm the significant increase in the expression of GAL and NPY in FB-positive neurons. Interestingly, previously described partial stomach resection experiment, independent of applied differential procedure, resulted in analogous changes in neuronal expression [52]. Indeed, these reports strongly suggest that sympathetic neurons while adapting to different pathological conditions respond with altered expression of bioactive substances.

It should also be noted that the results of the present study indicate the involvement of the sympathetic nervous system in pathological processes associated with gastric hyperacidity. However, further research is needed to understand the exact mechanism of the participation of active substances studied in lesions caused by hyperacidity. On the other hand, the immediate cause of the observed changes is also unknown. It may be due to direct chemical exposure to hydrochloric acid or its indirect action may trigger inflammation. The second explanation seems to be more likely due to inflammatory changes observed during gastroscopy examination.

## 5. Conclusion

The present data showed that experimentally induced hyperacidity triggered significant changes in the chemical coding of CSMG neurons projecting to the prepyloric area of the porcine stomach. The most remarkable differences include an upregulated expression of GAL and NPY and the* de novo* synthesis of nNOS and LENK as well as a downregulated expression of TH and D*β*H in the stomach-projecting neurons. These findings enrich existing knowledge of the participation of these active substances in adaptive mechanism(s) of the CSMG neurons during pathological processes within the gastrointestinal tract and suggest their potentially neuroprotective function. Thus, further studies should be carried out to elucidate the exact mechanism(s) of these changes during hyperacidity.
